# The distribution of benefits under China’s new rural cooperative medical system: evidence from western rural China

**DOI:** 10.1186/s12939-018-0852-7

**Published:** 2018-09-05

**Authors:** Sha Lai, Chi Shen, Yongjian Xu, Xiaowei Yang, Yafei Si, Jianmin Gao, Zhongliang Zhou, Gang Chen

**Affiliations:** 1 School of Public Policy and Administration, Xi’an Jiaotong University, No. 76 West Yanta Road, P.O Box 86, Xi’an, 710061 Shaanxi China; 2 Centre for Health Economics, Monash Business School, Monash University, Melbourne, Australia

**Keywords:** Health insurance, Equity, Benefit, China, New cooperative medical scheme

## Abstract

**Background:**

China’s New Cooperative Medical Scheme (NCMS) enables insured citizens to enjoy the same benefit package by paying a flat-rate premium. However, it still remains uncertain whether economically disadvantaged enrollees receive insurance benefits that at least match those of non-disadvantaged enrollees. This article, therefore, estimates the distribution of benefits under the NCMS across economic groups and compares the magnitude of economic-related inequity changes in the NCMS benefits.

**Methods:**

Data were drawn from two-wave large-scale representative and comparable cross-sectional household health survey datasets conducted in Shaanxi Province in 2008 and 2013. In total, 9506 (2008) and 38,010 (2013) NCMS enrollees were included. The benefits from the NCMS are measured in two ways: via the probability of receiving reimbursements and via the absolute amount of the obtained reimbursements. Two-part models were used to estimate the benefit distribution and to adjust benefits for health care needs. Concentration curve, dominance test of the concentration curve, and concentration index (CI) were used to estimate the overall degree of economic-related inequality. The degree of horizontal inequity was estimated via indirectly standardized measures based on the “equal treatment for equal needs” concept.

**Results:**

Our results indicate that economically affluent groups were more likely to receive reimbursements from the NCMS, and these reimbursements were also higher. Positive need-adjusted CIs for the probability of receiving reimbursements (CIs: 0.2027/0.1056 in 2008/2013) and the absolute amount of reimbursements (CIs: 0.3002/0.1660 in 2008/2013) further suggest the existence of clear pro-rich horizontal inequities in the benefits distribution under the NCMS. Encouragingly, a decreasing trend could be observed from 2008 to 2013, which suggests that horizontal inequities in NCMS benefits that favored the rich decreased over the investigated period, while the level of insurance benefits improved.

**Conclusions:**

Our study suggests that the benefits of NCMS are concentrated toward economically affluent groups. Although any trade-off between policy feasibility and equity has become a challenge for the formulation of social health insurance funding and benefit packages in developing countries, inequality can be gradually reduced through continuous adjustment of the medical insurance scheme, thus effectively targeting economically disadvantaged enrollees.

## Background

As one of three basic medical insurance schemes in China, the New Cooperative Medical Scheme (NCMS) is one of the most-widely covered health insurances in the world. It covers more than 800 million rural Chinese, which accounts for about 98.7% of the total rural population [1]. The scheme, which was started in 2003, aims to ensure that rural residents of China receive basic health care services, thus reducing the medical burden and achieving social fairness [2]. As a voluntary and governmentally organized scheme, it is largely financed through government subsidization and to a lesser extent through flat-rate household contributions. Unlike other social health insurance schemes with an income-related contribution (e.g., China’s Urban Employee Basic Medical Insurance), the NCMS is characterized by enabling insured citizens to enjoy the same benefit package including inpatient and outpatient by paying a flat-rate premium [3]. However, the equal policy design of the NCMS does not necessarily result in the equitable distribution of benefits.

As a means of risk-sharing, medical insurance protects the savings of enrollees from catastrophic medical costs. In addition, the income transfer in medical insurance may permit the purchase of health care that is beyond the consumer’s budget constraint and access to health care makes insurance very valuable [4]. Medical insurance achieved equitable financing and financial protection by levies insurance premium based on the enrollees’ ability to pay, while offering health services free of charge as the need arises [5]. Under the cost-sharing program (i.e., in deductibles, coinsurance, and copayments), the moral hazard effect of medical insurance can be reduced; however, the distribution of benefits may be undesirable [6]. The amount of the distributed benefits depends on both utilization and consumption of health care services, which are primarily driven by the demand for health care [7]. To a large extent, the use of services can be affected by budget constraints, i.e., enrollees with lower income have a smaller likelihood of consumption [8, 9]. Striking evidence indicates that a cost-sharing program leads to a pro-rich horizontal inequity in the distribution of health care services [10–12]. Hence, a flat-rate premium of the NCMS combined with the potential inequity of utilization [13–16] may result in an inequality of insurance benefit distribution.

Policy details of NCMS benefit packages play a role in balancing the distribution of benefits across an economic gradient. In terms of the NCMS policy design, different deductible and coinsurance rates were set based on the levels of medical institutions; here, a lower level of medical institutions indicates lower deductible and coinsurance rates. Table 1 shows the details of the NCMS design, using a county of western rural China as an example. Here, in 2013, deductibles ranged from 0 Yuan (1 US$ = 6.85 Yuan at current exchange rates) for township health centers (institutions at the lowest level that provide inpatient services) to 1500 Yuan for provincial hospitals, and reimbursement rates range from 90 to 100% for township health centers to 50% for provincial hospitals. These policy details are often considered to be positive because they indicate that seeking lower-level health care is likely to result in higher effective reimbursement. Different cost-sharing arrangements are partially designed to induce hesitation in enrollees before seeking health care and to forgo the use of services that are expected to result in a lower reimbursement rate. There is striking evidence that economically disadvantaged individuals, who were more sensitive to the price of health services were more likely to seek treatment in primary-level health-care facilities than economically advantaged individuals [7, 15]. Accordingly, whether and to what extent the distribution of benefits differs across the economic gradient under the NCMS in China requires close examination.

**Table 1:** Policy details of NCMS in 2008 and 2013

	2008	2013
Target population	Rural residents	Rural residents
Risk-pooling unit	County	County
Enrolment, %	90.1%	98.7%
Total premium per person (Yuan)	50	300
Government subsidy per person (Yuan)	40	250
Individual contribution (Yuan)	10	50
Benefit design		
Reimbursement for inpatient care		
Reimbursement ceiling (Yuan)	10,000 per capita	150,000 per capita
Deductible/reimbursement rate^a^ (Yuan/%)		
Township health centers	50/50–60%	0/90–100%
County hospitals	200–500/40–50%	200–500/70–80%
Municipal hospital	1000/30–40%	1000/60%
Provincial hospital	1500/20–30%	1500/50%
Reimbursement for outpatient care		
Reimbursement ceiling (Yuan)	NA (Outpatient pooling fund has not been established in 2008. It began in 2009.)	20,000 per capita
Reimbursement rate^a^	NA	70% in village clinics and 60% in township health centers.
Special disease of outpatient (Ceiling, Yuan/reimbursement rate^a^, %)	3 types of critical illness and 11 types of chronic diseases of outpatient. (1000/50%)	3 types of critical illness and 13 types of chronic diseases of outpatient. (20,000/60%)
Reimbursement method	Later reimbursement	Immediate reimbursement and later reimbursement

Note: (1) Policy details of the NCMS are illustrated using a county of western rural China as example. There is heterogeneity in policy details across counties because county governments retain discretion over the details

(2) ^a^The reimbursement rate is the percentage of the medical bill after meeting any deductibles and scope of reimbursement under NCMS

(3) *NA* data not available

Several empirical studies have attempted to explore the distribution of benefits of medical insurances among insured populations across different economic levels. For example, Huang et al. analyzed the net benefit distribution under Taiwan’s National Health Insurance program and reported that an apparent pro-poor pattern was observed under wage-based premiums; however, in terms of flat-rate premiums, such a pro-poor pattern was less obvious [17]. This study provides examples for the net benefit distribution of co-financing models by employees, employers, and governments. Pan et al. and Tian et al. analyzed the distribution of benefits under China’s Urban Resident Basic Medical Insurance (URBMI) targeting the urban population with an exception of formal sector employees; the authors reported that the rich benefit more from this particular insurance [18, 19]. Wang et al. analyzed the equity of benefits under the rural Cooperative Medical System (CMS) in China and concluded that under the “low premium and high coinsurance benefit design” model, regardless of the individual health status, high-income groups always benefit more from the CMS than low-income individuals [20]. These findings are not fully applicable to the NCMS. The insurance financing mode of Taiwan favors the poor, while the NCMS funding is equal for each insured citizen, which means that all enrollees pay the same premium regardless of their income. Several differences exist in the target population and benefit packages, cost-sharing arrangements, and financing modes between NCMS and URBMI or CMS [3, 21, 22]; therefore, analyzing the findings of the benefits distribution under URBMI and CMS is not fully generalizable for the NCMS. Few recent studies about insurance benefits have attempted to look into the magnitude of economic-related horizontal inequity or have ignored enhancement of NCMS altogether [23, 24].

The NCMS has undergone rapid enhancement and changes during recent years [25]. To equip the population with both affordable and equitable basic health care, the Chinese government unveiled a new Health Care Reform in 2009 [26]. Under this plan, the Chinese government enhanced its investment in basic medical insurance schemes. According to macroeconomic data, the expenditure of China’s government on the subsidy of medical insurance schemes reached 442.9 billion Yuan (roughly US$ 64.7 billion) in 2013, which amounts to 46% of the total government health expenditure [1]. This figure was close to three times the amount of 2008 [1]. The NCMS has improved with the growth of financing and coverage, which led to an enhancement of the reimbursement ratio and financial protection. Table 1 shows the changes of NCMS financing and benefit packages from 2008 to 2013. It became clear that, compared to 2008, the NCMS has greatly improved the financing and benefits package, i.e. higher reimbursement rates, higher reimbursement ceiling, expansion of the scope of reimbursement, simplification of the reimbursement method, and lower deductibles in township health centers. In the data used for this study, the ratio of actual out-of-pocket payment was approximately 50% of the total medical expenditures for NCMS inpatients in 2013, while it was 70% in 2008.

So far, empirical studies evaluating the equality in NCMS benefits under the background of the increasing benefit design and government subsidies of the scheme are limited. Theories associated with social health insurance indicate that the change of insurance generosity may affect the individual demand for health care by changing the out-of-pocket payment at the time of consumption, and furthermore affecting the distributional consequences [6, 7, 27]. Within the health area, the renowned RAND health insurance experiment of the 1970s investigated how copayment affects the use of health care services. Analyses of the RAND experiment reported that participants with lower income were more sensitive to cost-sharing than those with higher income; furthermore, participants with higher deductibles and coinsurance rates reduced their use and expenditures of health care services [8]. In addition, previous studies reported that simplification of the reimbursement procedures and expansion of the scope of reimbursement may improve health care utilization among the poor [13, 28]. Therefore, the distribution of benefits across economic gradients may be improved in response to the improved generosity of the NCMS.

The main purpose of this study was to investigate whether and to what extent the distribution of benefits differs across economic groups in 2008 and 2013; furthermore, whether and to what extent the magnitude of inequality in benefits changed under the background of increasing benefit design of NCMS was also investigated for the time-frame from 2008 to 2013. The equity of the basic medical insurance system is a central indicator to evaluate the New Health Care Reform [29]. Our results provide evidence for the further reform of the basic medical insurance in China and provide meaningful policy implications toward universal coverage of health insurance among other countries that face similar challenges.

## Methods

### Data sources

Data were drawn from two-wave large-scale representative and comparable cross-sectional household health survey datasets obtained in 2008 and 2013 as part of the fourth and fifth National Health Services Survey (NHSS) in Shaanxi Province. Shaanxi province is considered an economically underdeveloped region in western China. The NHSS questionnaire collected detailed individual demography data and socioeconomic information, health status, insurance status, health care utilization, and expenditure. Each household was interviewed face-to-face using a structured questionnaire.

A multi-stage stratified cluster random sampling method was used to collect representative samples for each wave. In each wave, a new set of participants was sampled from Shaanxi Province. In brief, in 2008, 44 counties in urban areas or districts in rural areas were randomly selected into the sample, while in 2013, 32 counties or districts were selected; among these, 75 townships in urban areas or sub-districts in rural areas were randomly selected in sampled counties or districts in 2008, while 160 were samples in 2013. Then, 150 villages or communities were randomly selected from townships or sub-districts in 2008, while 320 villages or communities were samples in 2013. Finally, 5960 and 20,700 households were interviewed in 2008 and 2013, respectively. In total, 18,290 (2008) and 57,529 (2013) residents were identified. The Myer’s Blended indexes were 1.67 and 1.63 for 2008 and 2013, suggesting that the respondents in each wave were representative for this region.

The NHSS was organized by the National Health Commission of the People’s Republic of China and presents the largest health survey in the area. During data collection, considerable quality control measures were implemented and have been described in detail before [22]. Based on a series of quality control measures, high response rates (> 85%) and high consistencies between survey and re-interviewed survey (> 95%) were achieved in all two-wave surveys.

In our analysis, we only focused on residents that were enrolled in the NCMS and were aged 15 or above. Children below 15 years of age were excluded from the sample because they could not answer several questions about socio-economic characteristics and health status. In total, 9506 (2008) and 38,010 (2013) NCMS enrollees were adopted for analysis in this study.

### Variables

The variables used in this study were classified into four categories: a) benefits obtained from the NCMS (outcomes), b) economic status, c) health care need factors, and d) other socioeconomic factors.

- Benefits from the NCMS

The benefits obtained from the NCMS were measured in two ways: 1) probability of receiving reimbursements; 2) the absolute amount of the obtained reimbursements. The probability of receiving reimbursements refers to the probability of receiving reimbursements of hospitalization expenses from the NCMS in the year before the survey. This variable was coded as yes = 1 and no = 0. The absolute amount of reimbursements was used to indicate the intensity of benefits, which captured the amount of hospitalization reimbursement received from the NCMS during the year before the survey.

(b) Economic status

The economic status variable used to rank the population was the household consumption expenditure per equivalent adult. It has been proposed that self-reported consumption expenditures are better indicators than the income to assess the household economic status in developing countries [30]. The following formula: AE = (A + αK)^*θ*^, was used to define the number of adult equivalents and was employed to derive the household consumption expenditure of an equivalent adult. In the formula, A and K represent the number of adults and children in the household, respectively, α represents the “cost of children” (equaling 0.3 in developing countries), and θ represents the scale of family economy (equaling 0.75 in developing countries) [31, 32]. For the regression analysis, the economic status variable was divided into five percentiles, where the first quintile represents the poorest economic group and the fifth quintile represents the wealthiest.

(c) Health-care need factors

The concept of “need” has received a variety of interpretations in relation to the definition of equity in health care delivery [33]. Consequently, the different health care utilization rates across individuals in different states of need are appropriate. In practice, economic-related inequity was measured by estimating the need-adjusted concentration index (CI) [34]. In this study, four variables (age, gender, presence of chronic conditions, and self-rated health) were used as proxies to adjust for differences in health care needs; these are commonly employed indicators of individual health status [35]. Age was categorized into three groups: 15–44, 45–59, and 60 or above. Gender was defined as male/female. The chronic conditions were defined as yes/no. The presence of chronic conditions in this analysis referred to the self-reporting of physician-diagnosed chronic diseases via the question “have you ever been diagnosed with any chronic diseases during the last six months?”. The reported chronic diseases were coded according to the International Classification of Diseases system, and mainly included, such as hypertension, diabetes mellitus, cardiovascular diseases, chronic obstructive pulmonary disease, musculoskeletal disease, mental disease, and cancer. Self-rated health was measured by using a visual analogue scale, which ranged from 0 (worst health state) to 100 (best health state).

(d) Other socioeconomic factors

Other socioeconomic variables included the educational level (primary or below, middle school, high school, and college or above), marital status (unmarried, married, and other), employment status (employed or unemployed), commercial medical insurance (yes or no), and time to the nearest health-care facility (less than 15 min, 15 min or above).

### Statistical analyses

#### Two-part models

Two-part models were used to investigate the benefit distribution of the NCMS for each wave separately. Two-part models are often used to model cost data that include many zero observations [36]. The first part of our study builds a model for the probability of receiving reimbursements and the second part builds a model for the absolute amount of the received reimbursements, which can be expressed as follows:

- The first part captures the difference between the enrollees who benefited from the NCMS and those who did not, specified as a Probit Model.

1 $$ P\left(\mathrm{Reimburse}=1\right)=\upalpha +{\sum}_j{\beta}_j{x}_{ji}+{\sum}_k{\gamma}_k{\omega}_{ki}+{\mu}_i $$

Where *P* represents the probability of receiving reimbursement. *x*_*j*_ represents a set of need factors including age, gender, chronic conditions, and self-rated health. *ω*_*k*_ represents a set of socioeconomic factors including economic status, educational level, marital status, employment status, commercial medical insurance, and time to the nearest health-care facility. The parameters β and γ represent the coefficients of independents.

(b) The second part captures the determination mechanism of the absolute amount of the reimbursement among those who received NCMS benefits. Since the distribution of the amount of the reimbursements has a skewed distribution, the dependent variables are transformed logarithmically. The log-transformed OLS models are as follows:

2 $$ \mathrm{Ln}\left(\mathrm{Reimburse}\_\mathrm{amount}/\mathrm{Reimburse}=1\right)=\upalpha +{\sum}_j{\beta}_j{x}_{ji}+{\sum}_k{\gamma}_k{z}_{ki}+{\mu}_i $$

The dependent is the logarithms of the absolute amount of reimbursements among those who received NCMS benefits. Slightly different from Eq. (1), *z*_*k*_ represents all variables in *ω*_*k*_ except for the time to the nearest health-care facility.

#### Methods to measure inequality and inequity

The concentration curve and concentration index are common measures to quantify inequalities in health and health care and both were used to summarize the overall magnitude of the economic-related inequality of NCMS benefits in our study. We also applied indirectly standardized measures to estimate need-adjusted CIs of the benefits distribution, assuming identical distribution of health care needs across economic groups, i.e. the degree of horizontal equity [37]. Moreover, dominance tests were used to test whether the concentration curve dominates (i.e., lies above) the 45-degree line and the Lorenz curve [34].

The concentration curve maps the cumulative proportion of the population, ranked by economic level from lowest to highest on the horizontal axis, against the cumulative proportion of benefits on the vertical axis. The 45-degree line of perfect equality reflects an equal distribution of NCMS benefits across the economic gradient. A curve above the 45-degree line indicates a pro-poor distribution i.e., that NCMS benefits are more highly concentrated amongst the poor, and vice versa. The degree of inequality increases as the concentration curve diverges from the 45-degree line.

The dominance test was performed to evaluate the distribution of the NCMS benefits by setting a target distribution. One alternative is to compare the distribution of the benefits with population shares (i.e., the 45-degree line). If the NCMS benefits were considered as part of the income of the enrollee, a further alternative is to compare the distribution of the benefits with the income distribution (i.e., the Lorenz curve). The latter requires that the concentration curve of the reimbursed amount dominates the Lorenz curve, which is obviously much less demanding than dominating the 45-degree line. A negative sign indicates that the 45-degree line/Lorenz curve dominates the concentration curve, while a positive sign indicates that the concentration curve dominates 45-degree line/Lorenz curve [34]. A multiple comparison approach was performed to test the dominance at the 5% significance level [34].

The CI is defined as twice the area between the concentration curve and the line of equality (the 45-degree line). The CI index can range from − 1 to 1 and an index of 0 is equivalent to perfect equality. A negative CI signifies a pro-poor distribution, while a positive CI signifies a pro-rich distribution. The CI formula is as follows:

3 $$ \mathrm{CI}=\frac{2}{\mu}\mathit{\operatorname{cov}}\left({y}_i,{r}_i\right) $$

Where y represents the NCMS benefits indexes; μ represents the mean of the NCMS benefits indexes, and r represents a fractional rank in the economic distribution.

In terms of the “equal treatment for equal needs” concept, benefits should be decided by the health care needs alone, and not by any other socioeconomic factors [17]. Here, we employed the indirect standardization method to estimate the degree of horizontal inequity, i.e., a need-adjusted inequality, by adjusting differences in health care needs. Such an indirect method of standardization has been suggested as a good alternative for measuring horizontal inequity [34]. We standardized benefit indexes by health care need as determined by age, gender, presence of chronic conditions, and self-rated health. A detailed method to conduct the standardization has been documented before [34, 38]. In brief, we used two-part models to generate the predicted probability and the amount of received reimbursements diminished the influence of health care need across economic groups; then, the CIs were calculated for these standardized variables. In addition, to calculate the predicted amount of reimbursement, the predicted log amount of reimbursements was re-transformed to a raw scale, using the smearing technique [39]. Control variables were included in the standardizing regression and results were found to be relatively insensitive to the inclusion of control variables.

Standard errors were adjusted for clustering at the village level in all models. *P* < 0.05 was considered statistically significant. All statistical analyses were conducted using the Stata software package, version 14.0.

## Results

### Descriptive statistics

Table 2 presents a summary of socio-demographic characteristics and health status for the samples of NCMS enrollees split by the year of survey. A total of 9506 (2008) and 38,010 (2013) samples were included in this analysis. With the exception of gender and economic quintiles, statistically significant differences were found in all characteristics across two waves (*P* < 0.05). In 2013, there were more respondents aged 45–59 years and 60 years or above than in 2008. Approximately 14.25% of the respondents reported the presence of at least one chronic condition in 2008, while this figure was 21.89% in 2013. In terms of self-rated health, respondents in 2013 obtained slightly higher scores than those in 2008. The majority of respondents had a middle school education or below, were married, and were employed in two waves. Only a small fraction of respondents purchased commercial medical insurance in 2008 (4.79%) and 2013 (4.00%). In 2013, 72.62% of the respondents reported that it takes less than 15 min to reach the nearest health-care facility, which was higher than in 2008 (66.06%).

**Table 2:** Characteristics of samples of NCMS enrollees in 2008 and 2013

Variables		2008	2013
Age(year)^†^, N (%)	15–44	4888 (51.42)	16,423 (43.21)
	45–59	2927 (30.79)	12,711 (33.44)
	60 or above	1691 (17.79)	8876 (23.35)
Gender, N (%)	Male	4653 (48.95)	18,424 (48.47)
	Female	4853 (51.05)	19,586 (51.53)
Chronic conditions^†^, N (%)	No	8151 (85.75)	29,689 (78.11)
	Yes	1355 (14.25)	8321 (21.89)
Self-rated health^†^, Mean (SD)		80.10 (13.40)	80.85 (13.37)
Economic status, N (%)	Poorest	1922 (20.22)	7603 (20.00)
	Poorer	1887 (19.85)	7678 (20.20)
	Middle	1904 (20.03)	7527 (19.80)
	Richer	1893 (19.91)	7601 (20.00)
	Richest	1900 (19.99)	7601 (20.00)
Educational level^†^, N (%)	Primary or below	4161 (43.77)	16,964 (44.63)
	Middle school	3977 (41.84)	15,646 (41.16)
	High school	1179 (12.40)	4387 (11.54)
	College or above	189 (1.99)	1013 (2.67)
Marital status^†^, N (%)	Unmarried	1807 (19.01)	4829 (12.70)
	Married	6884 (72.42)	29,960 (78.82)
	Other	815 (8.57)	3221 (8.47)
Employment status^†^, N (%)	Employed	6570 (69.11)	29,891 (78.64)
	Unemployed	2936 (30.89)	8119 (21.36)
Commercial insurance^†^, N (%)	Yes	455 (4.79)	1519 (4.00)
	No	9051 (95.21)	36,491 (96.00)
Time to the nearest health-care facility^†^, N (%)	Less than 15 min	6280 (66.06)	27,602 (72.62)
	15 min or above	3226 (33.94)	10,408 (27.38)
Total sample, N		9506	38,010

Note: ^†^Statistically significant differences (*P* < 0.05) were found in the characteristics across two waves using chi-square test or Students’ t-test

### Distribution of benefits

Table 3 shows the percentage of receiving reimbursements and the absolute amounts of reimbursements received from the NCMS by economic quintiles for both 2008 and 2013. In summary, 4.29% and 8.61% of the NCMS enrollees received reimbursements in 2008 and 2013, respectively; the amounts of received reimbursements per recipient were 1234.26 Yuan in 2008 and 4064.16 Yuan in 2013.

**Table 3:** Distribution of benefits by economic quintiles in 2008 and 2013

Economic status (Quintile)	2008		2013	
	Percentage of receiving reimbursements, % (95% CI)	Amount of the obtained reimbursements, Yuan (95% CI)	Percentage of receiving reimbursements, % (95% CI)	Amount of the obtained reimbursements, Yuan (95% CI)
Poorest	2.24 (1.58–2.90)	832.14 (622.07–1042.21)	7.34 (6.75–7.93)	3410.04 (2877.08–3943.01)
Poorer	3.07 (2.29–3.85)	748.19 (572.04–924.34)	7.97 (7.36–8.58)	2905.59 (2607.23–3203.95)
Middle	4.78 (3.82–5.74)	714.15 (586.16–842.15)	7.98 (7.37–8.60)	2934.18 (2656.89–3211.46)
Richer	4.23 (3.32–5.13)	884.84 (666.48–1103.20)	8.96 (8.32–9.60)	3947.16 (3464.38–4429.95)
Richest	7.16 (6.00–8.32)	2122.24 (1386.05–2858.42)	10.79 (10.09–11.49)	6299.32 (5659.49–6939.16)
Total	4.29 (3.88–4.70)	1234.26 (947.60–1493.91)	8.61 (8.33–8.89)	4064.16 (3836.44–4291.87)
Sample size	9506	408	38,010	3272

Clearly, the distribution of benefits under the NCMS showed a gap among different economic groups. Comparing the percentage of benefiting across economic groups showed that the higher economic quintiles are more likely to reap reimbursements. In terms of the amount of obtained reimbursements, the highest economic group received the highest amount of reimbursements, receiving about two times as much as the economically lowest groups. These results also indicated increases in the percentage of benefiting both in each economic group and in total from 2008 to 2013.

### Regression results

Table 4 shows the regression results with the marginal effects of the Probit Model and the coefficients of the OLS Model.

**Table 4:** Regression results using two-part models in 2008 and 2013

Variables	2008				2013			
	Participant (Probit)		Continuous (OLS)		Participant (Probit)		Continuous (OLS)	
	Coeff.	SE	Coeff.	SE	Coeff.	SE	Coeff.	SE
Age (Ref: 15–44 years)								
45–59	−0.011*	0.005	0.256	0.165	− 0.012**	0.003	0.173**	0.057
60 or above	−0.005	0.008	0.246	0.177	0.016 **	0.006	0.164*	0.064
Gender (Ref: Male)								
Female	0.015**	0.005	−0.315*	0.136	0.018**	0.003	−0.206**	0.040
Chronic conditions (Ref: No)								
Yes	0.056**	0.011	−0.262	0.143	0.089**	0.005	−0.011	0.045
Self-rated health	−0.001**	0.000	−0.009*	0.004	−0.002**	0.000	−0.011**	0.001
Economic status (Ref: Poorest)								
Poorer	0.009	0.007	−0.045	0.226	0.014**	0.005	0.022	0.066
Middle	0.028**	0.007	−0.118	0.148	0.018**	0.005	0.065	0.065
Richer	0.020**	0.007	0.018	0.155	0.028**	0.005	0.195**	0.072
Richest	0.049**	0.008	0.528**	0.170	0.045**	0.007	0.566**	0.080
Educational status (Ref: Primary or below)								
Middle school	0.006	0.006	−0.101	0.133	−0.001	0.004	0.009	0.049
High school	0.000	0.009	−0.134	0.183	−0.004	0.006	0.072	0.076
College or above	−0.005	0.017	−0.038	0.487	0.013	0.012	0.136	0.166
Marital status (Ref: Unmarried)								
Married	0.024**	0.005	0.106	0.222	0.050**	0.005	−0.288**	0.106
Other	0.014*	0.007	−0.029	0.281	0.026**	0.005	−0.349**	0.114
Employment status (Ref: Employed)								
Unemployed	−0.004	0.005	0.189	0.120	0.011*	0.005	0.033	0.051
Commercial insurance (Ref: No)								
Yes	−0.003	0.009	0.171	0.195	−0.007	0.009	−0.162	0.118
Time to the nearest health-care facility (Ref: Less than 15 min)								
15 min or above	−0.009	0.005			0.000	0.004		
Sample size	9506		408		38,010		3272	

Note: (1) Clustered robust SEs in parentheses; (2) Ref means Reference level; (3) *P < 0.05, **P < 0.01

In terms of the probability for receiving reimbursements, economic status, marital status, and health need variables were independently associated with the probability of receiving reimbursements both in 2008 and 2013. The employment status was found to be significantly associated with the probability of receiving reimbursements in 2013 rather than in 2008. Specifically, according the regression results for 2013, females, who were aged 60 years or above, suffered from chronic conditions, had lower scores of self-rated health, were married, divorced, or widowed, and were unemployed were more likely to receive reimbursements from the NCMS. Moreover, the results show that reimbursements were more likely to occur in higher economic groups.

Among the recipients of benefits, economic status, gender and self-rated health were significantly associated with the amount of received reimbursements both for 2008 and 2013; age, and marital status were significantly associated with the amount in 2013 but not in 2008. In comparison with the result for 2013, several factors were not significantly different with corresponding reference groups, which was possibly caused by limitations of the sample size. The results for 2013 show that, richer or richest economic groups among benefitted recipients received significantly more reimbursements; the benefitted recipients aged 45 years or above were likely to receive more reimbursements than those aged 15 to 44; males with lower scores of self-rated health and unmarried recipients received more reimbursements.

### Inequality analysis of benefits distribution

Table 5 shows the shares by economic quintiles, the CIs for the probability and amount of receiving reimbursements, and the dominance test of the concentration curve for both 2008 and 2013. The actual values and need-adjusted values of the probability and amounts of received reimbursements are listed in Table 5.

**Table 5:** Shares of NCMS benefits by economic quintiles, CIs, and dominance tests

Economic status (Quintile)	2008				2013			
	Actual percentage	Need-adjusted percentage	Actual amount	Need-adjusted amount	Actual percentage	Need-adjusted percentage	Actual amount	Need-adjusted amount
Poorest, %	10.54	10.13	7.11	7.16	17.05	14.87	14.31	14.08
Poorer, %	14.22	14.84	8.62	8.97	18.70	18.59	13.37	13.52
Middle, %	22.30	22.96	12.91	14.03	18.37	19.14	13.26	13.54
Richer, %	19.61	19.72	14.06	14.04	20.81	21.71	20.21	20.37
Richest, %	33.33	32.35	57.31	55.80	25.06	25.68	38.84	38.49
CI (SE)	0.2099** (0.0409)	0.2027** (0.0371)	0.3287** (0.0970)	0.3002** (0.0948)	0.0781** (0.0161)	0.1056** (0.0152)	0.1685** (0.0235)	0.1660** (0.0232)
Dominance test								
Against 45-degree line	–		–		–		–	
Against Lorenz Curve			None				+	

Note:(1) *P < 0.05, **P < 0.01; (2) Statistically significant differences (*P* < 0.05) were found in all values of CIs between 2008 and 2013 using the z-test. (3) - indicates the 45-degree line/Lorenz curve dominates the concentration curve; + indicates concentration curve dominates 45-degree line/Lorenz curve; none indicates non-dominance; a blank space indicates that the variable is not applicable

After adjusting for need variables, 32.35% of the NCMS reimbursements benefit recipients from the richest group, while 10.13% of recipients were found among the poorest group in 2008. However, for 2013, the percentages were 25.68% and 14.87% among the richest and poorest, respectively.

In terms of the amount of reimbursements, after adjusting for need variables, the richest group received 55.80% of the total reimbursed amount, while the poorest group received only 7.16% in 2008. In 2013, the shares of reimbursed amount were 38.49% and 14.08% among the richest and poorest group, respectively.

In 2008 and 2013, the values of CIs were all positive and statistically significant, which further suggests the existence of clear pro-rich inequities in the benefits distribution under the NCMS. After controlling for differences in health needs across economic groups, the values of CIs slightly changed in magnitude; however, a pro-rich distribution remained. The CI values for both actual or need-adjusted probability and the amount of received reimbursements were significantly smaller (*P* < 0.05) in 2013 than in 2008, suggesting that compared to 2008, the pro-rich bias softened slightly in 2013.

**Fig. 1:**
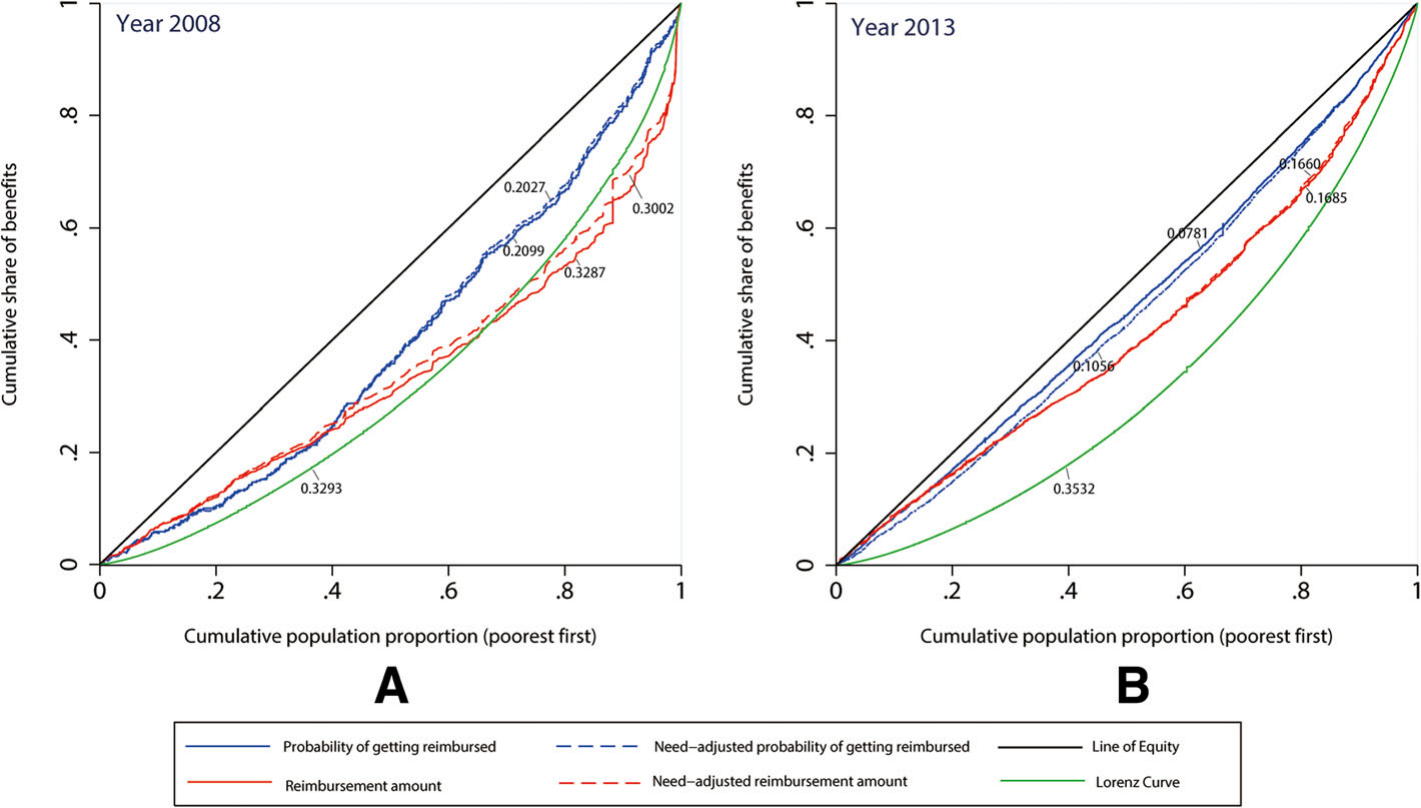
Concentration curves of NCMS benefits in 2008 and 2013. The concentration curves for unadjusted and need-adjusted probabilities and the amount of received reimbursements in 2008 are shown in panel A. The concentration curves for unadjusted and need-adjusted probabilities and the amount of received reimbursements in 2013 are shown in panel B

Figure 1 presents the concentration curves for the overall probability and amount of received reimbursements, as well as the Lorenz curve for household expenditure. Figure 1 shows that the 45-degree line remained above and dominated the concentration curves for probabilities and amount of reimbursement in 2008 and 2013 (Table 5), indicating a pro-rich inequity in the distribution of benefits under the NCMS. However, the concentration curves for 2013 were closer to the line of perfect equality, suggesting that in 2013, the distribution of benefits showed less economic-related inequity than in 2008. Moreover, the concentration curve for the amount of reimbursements dominated the Lorenz curve in 2013, indicating that the NCMS reimbursements were inequality-reducing relative to the income benchmark in 2013.

## Discussion

Although the goal of the NCMS is to provide equal financial protection for all enrollees, the distribution of benefits between the rich and the poor is not necessarily equitable. In this study, using two variables as proxies to measure benefits, i.e., the probability of receiving reimbursements and the absolute amount of received reimbursements, we assessed the distribution of NCMS benefits. This was conducted under the background of the increasing benefit design of the NCMS, using two-wave cross-sectional household health surveys conducted in 2008 and 2013. We found that the benefits of the NCMS are concentrated among higher economic groups; however, horizontal inequities in the NCMS favorably benefiting the rich decreased over the investigated period, while the level of insurance benefits increased.

Specifically, the regression results firstly show that, compared to the poor, the rich benefit more from the NCMS. These results are consistent with recent studies, showing that insurance benefits were more directed to the rich under the Urban Residents’ Basic Medical Insurance in China [18, 19]. The analysis of inequality further indicates clear pro-rich horizontal inequity in benefits distribution under the NCMS in both 2008 and 2013. Moreover, the inequality-reducing effect of NCMS reimbursements relative to the income benchmark was observed in 2013. After controlling for differences in health needs across economic groups, the obtained values of CIs slightly changed. The relatively small difference between unadjusted and adjusted CIs may be due to the relative equilibrium distribution of health need variables across an economic gradient. Comparing the degree of inequity between 2008 and 2013 showed that need-adjusted CIs for both probability and amount of receiving reimbursements in 2013 were significantly lower than in 2008. This indicates that the pro-rich inequality of the NCMS decreased. The trends of inequality in benefits distribution observed in this study are consistent with the trends reported in previous studies on inequality in access to health care under the NCMS [13], suggesting that the inequity in inpatient utilization decreased along with the improved generosity of the NCMS.

The regression results indicate that health need factors were independently associated with the probability and amount of receiving reimbursements. Females were more likely to receive reimbursements partly due to hospitalized delivery. The lower amount of reimbursements among female enrollees may be associated with their lower medical consumption caused by the lower social and economic position of females in rural China. In addition, the results indicated that marital status was significantly associated with both the probability and the amount of received reimbursements, which was consistent with previous observations on health services utilization [40]. Our study did not find a significant correlation between the presence of commercial medical insurance and NCMS benefits. In China, very few individuals of the rural population purchased commercial medical insurance and no adverse selection was apparent [41].

This distribution of benefits is the most direct result of the NCMS. In terms of the “equal treatment for equal needs” concept, NCMS benefits should be decided by the health care needs alone, and not by any other factors such as the enrollees’ economic level; consequently, individuals with the same health care needs should receive the same benefits. However, the obtained findings indicate that the NCMS favors the rich. The underlying reasons for this inequity are mainly the inequity of the enrollees’ health care utilization and the policy design of the NCMS in the financing and benefit package.

The launch of a social medical insurance is considered a crucial step to reduce inequality regarding the access of health care. However, evidence of developing countries indicates that voluntary public insurance schemes, particularly those with high premiums and coinsurance rates, may have little impact for the improvement of the health services access for the poor [42, 43]. Theoretical models in the literature predicted that price elasticity of the demand for health services is likely to be higher for individuals at the lower end of the economy [44]. Therefore, the cost-sharing program with a higher coinsurance is unlikely to reduce the discrepancies of health care utilization between the rich and the poor or promote their equity. As suggested by Nyman’s theoretical research on the value of health insurance, health insurance can help low-income enrollees to surpass budgetary constraints to access health care; however, the degree of access depends heavily on the insurance payment system [4]. Numerous empirical studies reported a significant positive correlation between the access to health services and the socioeconomic status of enrollees. Studies in developed countries showed that high-income individuals are more than twice as likely to use medical services compared to those with low income [45]. Reports from developing countries also suggested that the rich access more health services [46]. It seems that NCMS contributed less to reducing income-related inequality in health service utilization and the relative increase in utilization of hospitalization was higher among rich enrollees [14, 15, 47].

The NCMS has been designed to collect flat-rate premiums and to provide an equal package of benefits for all enrollees, regardless of their ability to pay. Contributions toward the financing of social insurance may redistribute income [34]. However, with regard to flat-rate premiums, the economically disadvantaged have to pay the exact same premium as all others; therefore, health insurance contributions comprise a decreasing share of income with increasing ability to pay. Although previous publications of theories and empirical evidence suggested that income-based (a progressive premium method) social insurance schemes tended to have a pro-poor pattern in the distribution of net benefits [5–7, 17], it is difficult to adopt a wage-based or income-based insurance program for rural residents without well-defined income or economic conditions. The NCMS covers such a large number of enrollees that it is also difficult to identify poor or vulnerable groups; therefore, a uniform standard of benefits package for all has been adopted.

Although the policy design of the system can achieve only limited effectiveness in achieving fairness, the NCMS is trying to improve and adjust. Compared to 2008, the NCMS has greatly improved the benefits package after the new medical reform. Correspondingly, in our results, the pro-rich inequality in benefits have decreased for 2013. The improvement of the NCMS is characterized by higher reimbursement rates, higher ceiling, expanded scope of reimbursement, simplified reimbursement method (for example, immediate reimbursement, i.e., patients can be compensated immediately after paying medical expenses), and lower deductibles in the primary medical institution. When the level of insurance package is high, medical expenditure is less dependent on the personal economic conditions [8]. In this case, for disadvantaged enrollees, enhanced access to health services increases the opportunities to receive benefits from the NCMS. Recent findings regarding access to health care showed that hospital admissions increased from 6.8% in 2008 to 8.4% in 2011 in rural areas in China, and inpatient reimbursement rates likewise increased from 32.9% in 2008 to 43.7% in 2011 [22].

The simplified reimbursement process may play an important role for the reduction of inequality in NCMS benefits. Specifically, the method of immediate reimbursement was used more often than before under the NCMS. For the disadvantaged economic population, later reimbursement, i.e., paying the full hospitalization cost first and then receiving reimbursements later, may cause current and future income difficulties for their family. The poor with need for treatment are therefore more likely to refuse treatment when expecting high health care costs. Immediate reimbursement, especially for the poor, can partially alleviate these current economic constraints and may therefore increase healthcare utilization [28].

Another shift in the NCMS policy was that the level of insurance benefits for lower-level medical facilities was greatly improved. For township health centers, deductibles were even removed, and reimbursement rates were raised to 90% or 100%. Numerous findings show that the poor were more likely to seek treatment in primary-level health-care facilities [48]. This design has lowered the threshold and economic burden for seeking treatment for enrollees with low economic background, thus benefitting the poor who need healthcare services from the medical insurance.

Furthermore, over time, the NCMS system has been progressively extended, increasing policy awareness confidence and gradually improving the status quo bias (i.e., the original habit of medical service utilization still adhered to in the case of insurance) among poor enrollees [49]. Thus, more health care needs may be released. In addition, other reforms within the new medical reform, such as the national essential medicines program, the establishment of a primary healthcare system, and a defined package of basic public health services for the population, may also play a facilitating role for the alleviation of the current inequality in NCMS benefits [50].

Finding the appropriate trade-off between policy feasibility and equity has become a challenge for the formulation of social health insurance funding and benefit packages in developing countries. However, this inequality can be gradually reduced through adjustments of the medical insurance scheme. For example, one promising adjustment could be the introduction of more favorable reimbursement methods, e.g., where the enrollees do not have to pay the full cost for each treatment in advance but only have to pay for the remaining portion after reimbursement. Currently, this approach is only implemented by China’s Urban Employee’s Basic Medical Insurance. This may further eliminate economic obstacles, especially for the poor. Another possible improvement might be to set distinct standards in premiums and benefit packages for vulnerable groups who are easily identified, such as the elderly and minimum living guarantee households.

To assure equitable access to health care and benefits distribution, insurance coverage alone is insufficient. The implementation of further relevant health policies can also be beneficial for reducing inequality in health insurance benefits. For instance, when the capacity and scope of primary health care facilities are improved, the disadvantaged may obtain added access to better medical services from primary health care facilities, which have lower deductibles and coinsurance rates.

This study has several limitations that require careful consideration. First, only four observable variables (age, sex, chronic conditions, and self-rated health) were used to adjust for health care needs; however, there may be other unobservable health statuses. Additionally, if the socioeconomic gradients disparities of the four health need variables are not homogenous, the socioeconomic disparities in health care needs may be weakened [51]. Second, we have been limited to repeat cross-sectional analyses. Longitudinal analysis of enrollees would allow increased scope for changes in economic-related inequity of benefits. Third, the quality of certain health-care services offered to identical patients may have varied between population groups; however, this study did not control for confounding variables related to the quality of health services as it was not feasible with the given large-scale household survey data. Further studies on insurance benefits could take the possible differences in the quality of health services received by enrollees into account across economic groups. Fourth, children below the age of 15 were excluded from this analysis due to their lack of available information regarding the required socio-economic characteristics and health status. Furthermore, outpatient reimbursement was not included as part of the NCMS benefits in this analysis, since the reimbursement information of outpatients is not available in our data neither for 2008 nor for 2013. These limitations could possibly lead to an underestimation of the benefits from NCMS for all enrollees and potentially lead to a bias of the results in terms of the distribution of benefits. However, this bias could be limited because the outpatient pooling fund of NCMS began in 2009 in the sample area and accounted for only a small part of the total pooling fund of the NCMS (less than 20%) in 2013. Despite these limitations, the results of this study provide the most realistic reflection of the benefits distribution and its inequities under the NCMS of western rural China.

## Conclusions

This study forms a first step towards evaluating the magnitude of inequities in the NCMS benefits in western rural China during different periods (before and after the New Health Care Reform). The NCMS, which is characterized as a flat-rate premium and equitable benefit package, led to pro-rich horizontal inequity in the benefits distribution in both 2008 and 2013. A reduced pro-rich inequity in the NCMS benefits was observed toward 2013; however, it may be difficult to achieve the equity goals without a more comprehensive policy design for the NCMS that specifically and effectively targets disadvantaged enrollees. For developing countries, which attempt to extend social health insurance coverage using government subsidies, our results provide evidence for further reforms and bears meaningful policy implications.

## Abbreviations

CI: Concentration index
CMS: Cooperative Medical System
NCMS: New Cooperative Medical Scheme
URBMI: Urban Resident Basic Medical Insurance
